# Supplementary material for: New Lead Schiff Bases Predominantly Mediate Vasorelaxant Activity Through α1 Receptor Blocking Activity
Source: Biomolecules. 2025 Apr 23;15(5):611. doi: 10.3390/biom15050611 (PMC12109045; doi:10.3390/biom15050611)
Supplement: Supplementary file 1 [file biomolecules-15-00611-s001.zip › biomolecules-3546006-supplementary.pdf]

Supplementary files showing synthesis and related spectral information of SB1 and SB2 molecules.

Synthesis of (S,E)-N-(4-methylbenzylidene)-1-phenylethanamine (SB1)

(S, E)-N-(4-methylbenzylidene)-1-phenylethanamine (SB1) was synthesized by reacting (S)-(-)-α Methylbenzylamine (1mmol) with 4-Methylbenzaldehyde (1mmol).

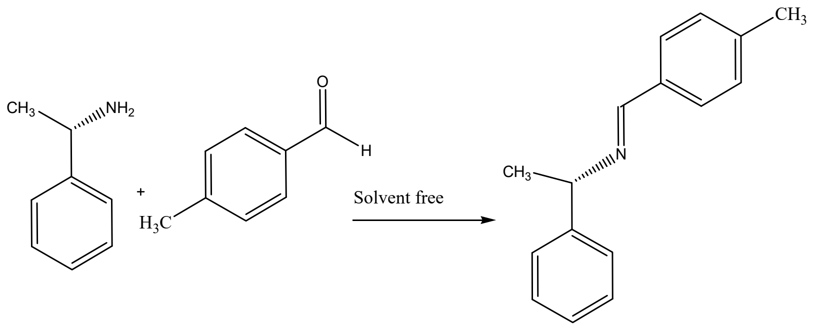

Synthesis of SB1

Figure S1:Chemical reaction pointing to the synthesis of compound (SB1). Molecular formula: ($C_{16}H_{17}N$)

Synthesis of (R,E)-N-(4-methylbenzylidene)-1-phenylethanamine (SB2)

(R, E)-N-(4-methylbenzylidene)-1-phenylethanamine (SB2) was synthesized by reacting (R)-(+)-α-Methylbenzylamine (1mmol) with 4-Methylbenzaldehyde (1mmol)

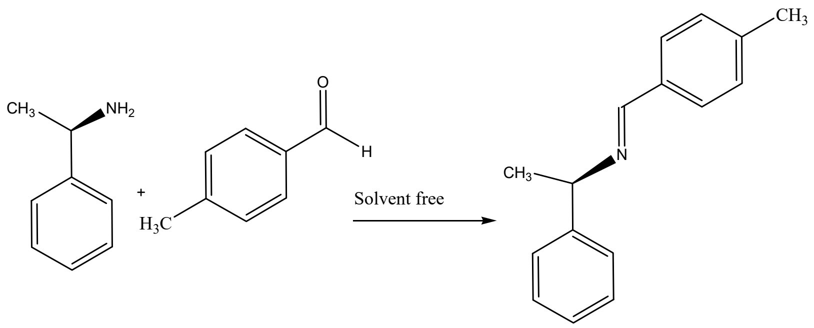

Synthesis of SB2

Figure S2:Chemical reaction pointing to the synthesis of compound (SB2). Molecular formula: ($C_{16}H_{17}N$)

Both chiral Schiff bases (SB1 and SB2) were synthesized by putting the reactants in mortar and pestle and grinded for 30 minutes without the addition of any solvent. Reactions were monitored by TLC. After completion, the products were filtered, washed with water, dried and recrystallized in ethanol. The yield of SB1 was 88.4 % and the yield of SB2 was 86 %.

Both compounds (SB1 and SB2) were soluble in chloroform and ethanol. HNMR showed doublet at 1.64 (SB1) and 1.62 (SB2) respectively confirms methyl group and singlet at 8.37 confirm the presence of azomethine proton.

IR spectra of compounds showed a strong band at 1487.12cm^{-1} (figure supplementary data) that attributed to imine ν (-CH=N) stretching [1].

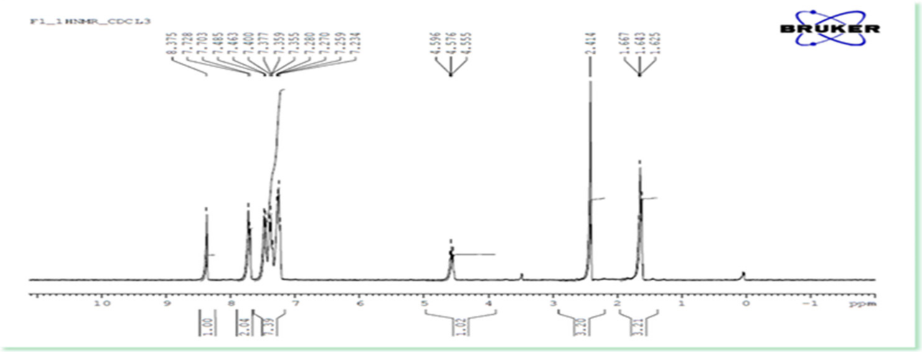

Figure S3: HNMR spectra of SB1

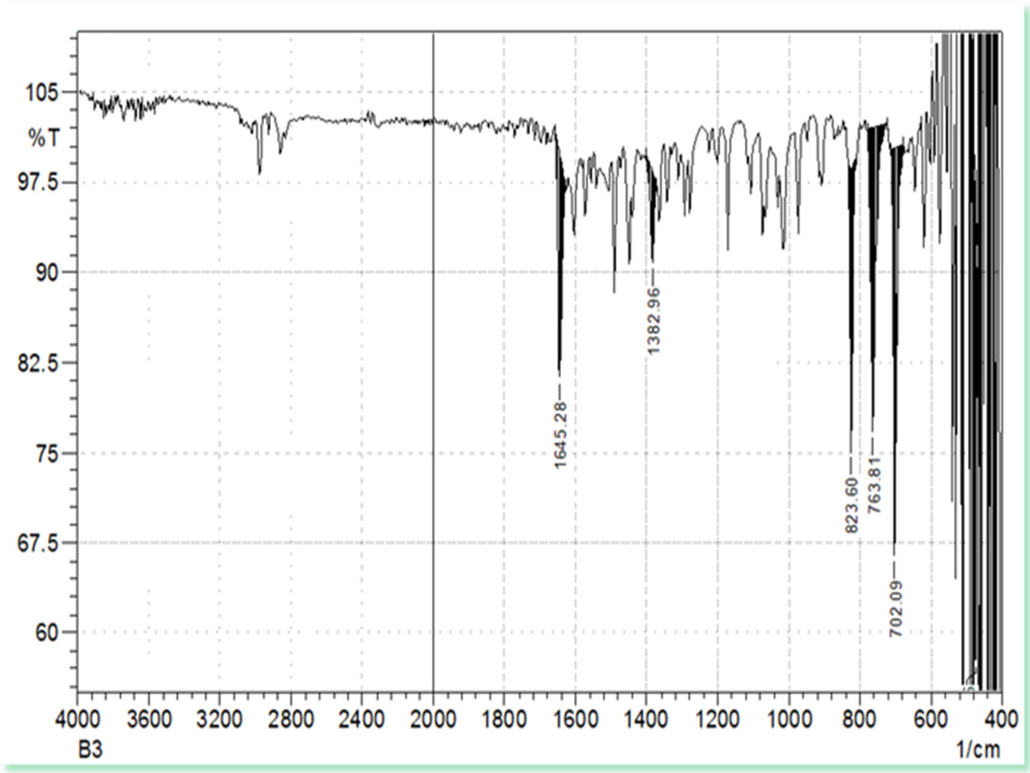

Figure S4: IR spectra of SB1

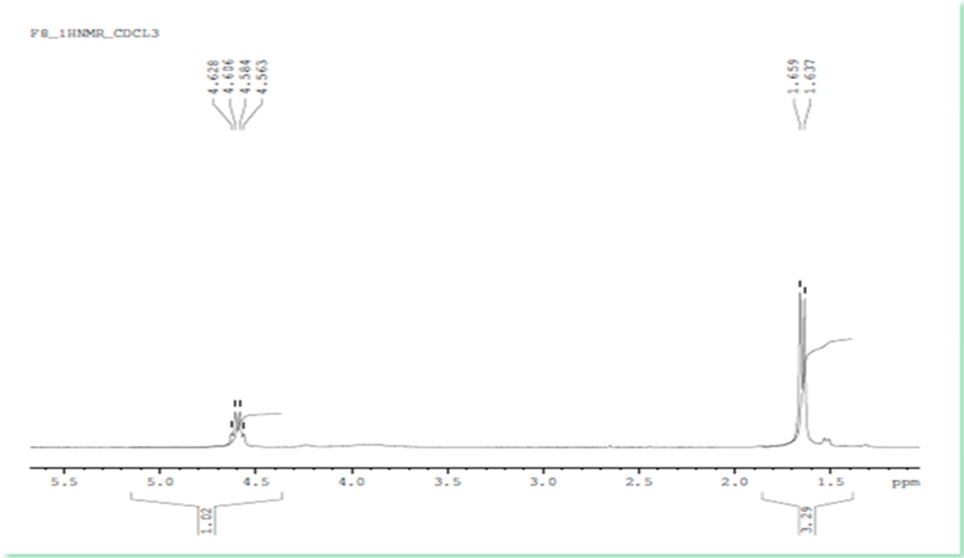

Figure S5: HNMR spectra of SB2

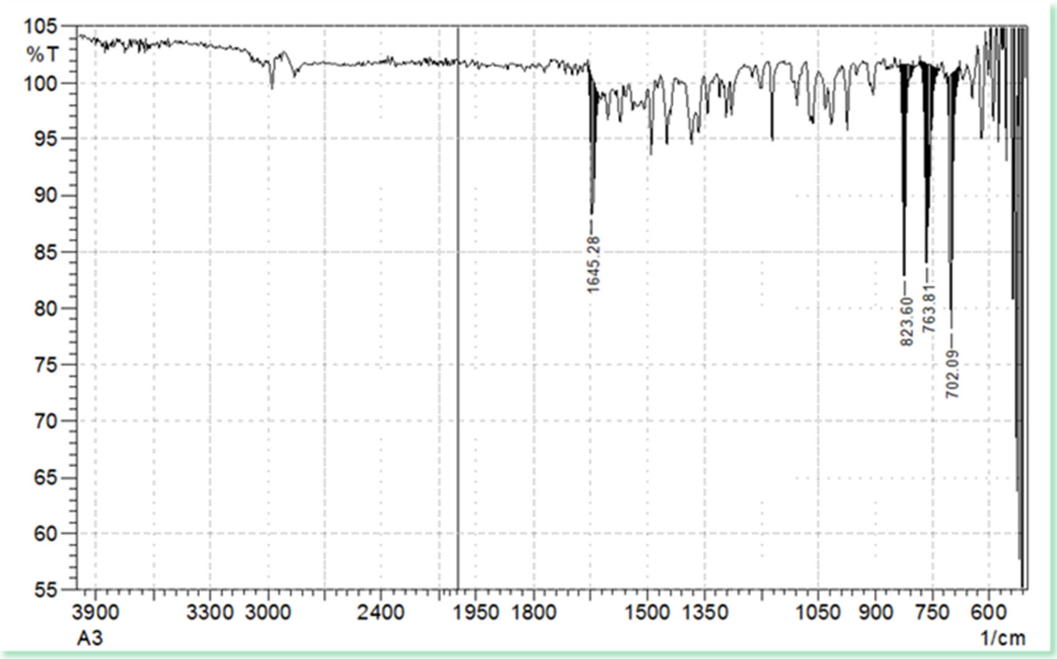

Figure S6: IR spectra of SB2

Reference:

Afridi HH, Shoaib M, Al-Joufi FA, Shah SWA, Hussain H, Ullah A, et al. Synthesis and investigation of the analgesic potential of enantiomerically pure schiff bases: a mechanistic approach. Molecules. 2022;27(16):5206.
